# Mediating effect of social anxiety on the association between self-esteem and internet addiction among Chinese vocational school students

**DOI:** 10.3389/fpubh.2025.1412480

**Published:** 2025-03-25

**Authors:** Yunjiao Zhu, Guifang Jin, Haiyan Shi, Chenyu Sun, Hongyuan Wei, Linsheng Yang, Jiahu Hao, Ying Sun, Puyu Su, Xiaoyan Wu, Xiaowu Tang, Zhihua Zhang

**Affiliations:** ^1^Shangcheng District Center for Disease Control and Prevention (Shangcheng District Health Supervision Institute), Hangzhou, Zhejiang, China; ^2^Department of Epidemiology and Biostatistics, School of Public Health, Anhui Medical University, Hefei, Anhui, China; ^3^Department of General Surgery, The Second Affiliated Hospital of Anhui Medical University, Hefei, Anhui, China; ^4^Anhui Provincial Center for Disease Control and Prevention, Hefei, Anhui, China; ^5^Department of Maternal, Child and Adolescent Health, Anhui Medical University, Hefei, China; ^6^Department of Medicine, Hefei Technology College, Chaohu, Anhui, China

**Keywords:** adolescents’, internet addiction, self-esteem (SE), social anxiety (SA), mediating effect

## Abstract

**Introduction:**

In today’s digital age, concerns about internet addiction among adolescents have escalated alongside the widespread use of the internet. Simultaneously, research has spotlighted the influence of psychological factors like self-esteem and social anxiety on addictive behaviors. The aim of this study was to verify the hypothesis regarding social anxiety as a mediator in the association between self-esteem and internet addiction.

**Methods:**

A total of 10,158 participants were randomly selected from five vocational schools in Anhui Province, China, with a mean age of 18.5 years. They completed a series of self-administered questionnaires, including the Young Internet Addiction Test (IATS), the Liebowitz Social Anxiety Scale (LSAS), and the Self-Esteem Scale (SES). Structural equation modeling (SEM) was employed to examine the mediating role of social anxiety between self-esteem and internet addiction, adjusting for demographic variables such as age, gender, and parental education.

**Results:**

The correlation analysis revealed that self-esteem was significantly negatively correlated with internet addiction, while social anxiety was significantly positively correlated with internet addiction. The indirect effect of self-esteem on internet addiction through social anxiety was 0.11 (*p* < 0.01), constituting 28.35% of the total effect. Additionally, the total impact of self-esteem on internet addiction was 0.278 (*p* < 0.01). Subgroup analyses by age and gender confirmed the robustness of these findings, with significant total effects observed across different age groups (14–18 years: total effects = 0.637; 18-24 years: total effects = 0.744; *p* < 0.01) and genders (male: total effects = 0.385; female: total effects = 0.744; *p* < 0.01).

**Discussion:**

The results indicate that social anxiety plays a significant mediating role in the relationship between self-esteem and internet addiction, affecting both directly and indirectly this association. These findings underscore the importance of addressing self-esteem and social anxiety in interventions aimed at reducing internet addiction among adolescents, suggesting that targeted psychological support could be pivotal in mitigating the risk of developing addictive behaviors online.

## 1 Introduction

A new type of addiction prevalent among individuals with extensive internet access poses a significant challenge in the information age. As evidenced, an increasing number of individuals, especially teenagers, exhibit addiction to the internet. Internet Addiction (IA) is known by various terms including Internet Addiction Disorder (IAD), Problematic Internet Use (PIU), Compulsive Internet Use (CIU) and Excessive Internet Use (EIU) (1). Currently, a uniform definition of internet addiction remains absent in the academic community, with various scholars have put forward different views (2). In this study, we have adopted a broader concept of IA, encompassing an individual’s excessive and uncontrollable use of computers, smartphones, or other digital devices to engage in various online activities (including online gaming, social media interactions, e-commerce, and smartphone usage). This compulsive usage pattern results in psychological and physiological impairments, which may subsequently lead to significant difficulties in social relationships, academic performance, or occupational functioning (3, 4). According to the report on internet addiction among Chinese teenagers, released by the Blue Book of Youth: Report on Internet Use by Chinese Minors (77) reveals a penetration rate of 99.2% among this demographic. And survey results on internet addiction among teenagers indicate that the proportion of teenagers with internet addiction accounts for approximately 14.1% according to Zhang et al. (4). Meanwhile, evidence suggests that similar situations are prevalent in other countries. A systematic review and meta-analysis revealed that the pooled prevalence of internet addiction among African college and high school students was 34.93%. Nazir S Hawi and Cecilie Schou Andreassen, among others, have both stated that with the social changes and the rapid development of science and technology in the current society, individuals, face various psychological health issues such as sleep problems, declines in interpersonal relationships, and low self-esteem, all stemming from internet addiction, which cannot be ignored (5, 6). Given the high incidence of internet addiction and the severity of its consequences, close attention should be paid to this significant issue.

In order to explore the psychological mechanisms behind internet addiction, this study adopts Cognitive Behavioral Theory (CBT), which emphasizes the role of cognitive and behavioral activities in an individual’s physiology and psychology. Compared to other social work theories (7), CBT places greater emphasis on the effective interactive relationship between the intrinsic cognitive levels of adolescent individuals and their surrounding social environment. In most cases, adolescents’ cognition and evaluation of their own behavior tend to be rational, leading to specific behaviors based on certain cognitions. However, when an individual’s self-cognition and evaluation are irrational, it can lead to adverse emotional responses and correspondingly irrational behaviors. Currently, CBT has been used to study internet addiction in different countries and groups. For instance, Chen et al. (8) investigated the impact of COVID-19 victimization experiences on college students’ mobile phone addiction based on future anxiety within the framework of CBT. Hu et al. (9) based on social cognitive theory and gender differences, validated a regulatory mediation model to explore the relationship between COVID-19 related stress and social networking addiction. These studies indicate that CBT provides a powerful theoretical framework for understanding internet addiction, helping to reveal how individual intrinsic cognition and external environment jointly contribute to the development of addictive behaviors.

Social anxiety (SA) is defined as “a marked fear or anxiety in one or more social situations where individuals believe they may be scrutinized by others” (10), social anxiety symptoms have been linked to attention biases towards threat-related social situations in adolescents (11). SA patients exhibit a strong aversion to social environment stimuli or threats (12). In social situations, individuals with social anxiety tend to direct their attention inwards, monitoring themselves and their performance, mistakenly using anxious feelings and negative self-images as evidence to support their negative beliefs (13). SA poses a significant public health and mental health concern among adolescents, not just exclusive to adults and the older adult (14). Several studies have confirmed the association between SA and IA (15–17). With previous research proposing that SA may be an etiological factor of IA among adolescents (18). Causing that individuals who experience SA are more likely to suffer from a heightened sense of loneliness (19). Consequently, they may turn to the internet to alleviate or relieve their face-to-face anxiety. As a result, they may spend increasing amounts of time online to alleviate their social anxiety. When internet usage becomes excessive, these individuals may become more dependent on network communication, and may increasingly avoid face-to-face communication. If adolescents are caught in such a cycle, it may cause more serious internet addiction and more severe social anxiety (20).

Self-esteem (SE) refers to a person’s self-acceptance, derived from an assessment of global self-worth, attractiveness, ability, and ability to achieve one’s own ambitions (21). Adolescence is a critical period for the formation of self-esteem in teenagers, representing a pivotal stage in their overall development (22). During this period, adolescents may face various pressures from academic performance, family harmony and social environment problems. If adolescents do not handle these problems well, they will have a negative impact on the formation of their self-esteem, and they will have negative emotions, forming low self-esteem easily (23, 24). SE includes self-affirmation, self-doubt and self-denial of adolescents (25). Children and adolescents are more susceptible to poor SE than adults. In turn, low SE can also affect the emotions of adolescents, leading to self-doubt and self-denial, thereby fostering fear and avoidance of social interaction (26). It has been suggested that poor SE can lead to various psychological issues. Adolescents with high self-esteem tend to have a more positive view of themselves and exhibit more initiative in interpersonal interactions. In contrast, those with low self-esteem are inclined to have a more negative self-evaluation and are more passive in social situations (27). Previous studies have shown that a person’s SE may be an important factor in the development of SA, which, in turn, may further deflate SE (28, 29). Low self-esteem is associated with a range of emotional and behavioral challenges, negative attitudes towards life (30), increased risk of depression (31), and potential addiction (32, 33). Based on the association between SE, SA, and IA, we propose hypothesis 1 (H1): low self-esteem is directly and positively related to internet addiction; and hypothesis 2 (H2): low self-esteem will be directly and positively related to social anxiety disorder.

Based on the previous studies (34, 35), addictive behavior is a reaction in which individuals experience stress stemming from social environment. It is reasonably assumed that teenagers who experience socially anxiety caused by low self-esteem may excessively use the internet coping mechanism to manage life stress and regulate negative emotions. Excessive usage of the internet will make adolescents more fearful of avoiding face-to-face communication, more sensitive to the stimulation of the social environment, and further damage their self-esteem. Finally, adolescents may become trapped in this cycle. This circle not only results in serious social anxiety, but also contributes to more severe internet addiction. Therefore, we proposed hypothesis 3 (H3): social anxiety partially mediates the relationship between low self-esteem and internet addiction.

To date, however, few studies have assessed the mediating effect of social anxiety on the association between internet addiction and self-esteem. This study advances existing knowledge by employing structural equation modeling (SEM) to examine the mediating role of SA between self-esteem and internet addiction, providing a new perspective on how self-esteem influences adolescents’ internet behavior. Additionally, our study’s representative sample of adolescents from five vocational schools in Anhui Province, because of the potential vulnerabilities of vocational school students, who often have greater internet accessibility and potentially lower self-control capabilities, are considered to be at higher risk for internet addiction (36, 37). By focusing on this population, we could provide a more nuanced understanding of how social anxiety might influence the relationship between self-esteem and internet addiction. By validating the direct link between SA and internet addiction and exploring how low self-esteem contributes to internet addiction through SA, our findings deepen the understanding of the psychological mechanisms behind internet addiction and provide a theoretical basis for prevention and intervention strategies.

Based on the reason, we conducted a large-scale, cross-sectional study among adolescents of vocational school students in Anhui province. To achieve this, this study has been designed with the following three steps: (1) to investigate the prevalence of internet addiction and the general demographic characteristics of adolescents with internet addiction; (2) to explore factors related to internet addiction; and (3) to test the three hypotheses: Hypothesis 1 (H1): Low self-esteem is directly and positively associated with internet addiction (IA). Hypothesis 2 (H2): Low self-esteem is directly and positively associated with Social Anxiety (SA). Hypothesis 3 (H3): Social anxiety partially mediates the relationship between low self-esteem and internet addiction. Specifically, the central aim of this study was to investigate the mediating role of social anxiety in the relationship between self-esteem and internet addiction among adolescents based on cognitive behavioral theory. Our primary objective was to determine whether social anxiety acts as a mediator in the link between self-esteem and the risk of internet addiction, a relationship that has become increasingly pertinent in the digital age. To ensure the robustness of our study findings, we controlled for key demographic variables in our mediation model based on the results of preliminary analyses of demographic differences. These variables, including age, gender, and parental education, were selected based on their theoretical importance in adolescent psychological development and internet use behavior. For instance, age (38, 39) and gender (40) have been shown to influence the prevalence and severity of internet addiction, while parental education affects adolescents’ access to the internet and their ability to manage internet use (41). Our analysis focused on examining the direct and indirect effects of self-esteem on internet addiction, with social anxiety serving as a potential mediating variable. By adjusting for these key variables in our analysis, we were able to gain a deeper understanding of these complex interactions. To achieve this, our analysis focused on examining the direct and indirect effects of self-esteem on internet addiction, with social anxiety as the potential mediator. We adjusted for key demographic variables in our analysis to ensure a robust understanding of these complex interactions.

## 2 Materials and methods

### 2.1 Participants

In this cross-sectional study, participants were recruited from vocational schools, including vocational high schools and advanced vocational colleges, located in five cities of Anhui province: Chuzhou, Fuyang, Chaohu, Tongling, and Anqing. In each school, all first-grade students were invited to participate in the survey. We employed a stratified sampling method to ensure that our sample is representative of the student population from vocational and technical schools in different regions. Initially, we calculated the preliminary sample size based on the stratified sampling formula, and then we increased it by 10% to account for potential non-response.


n=z∝22∑Ni2Pi1−Pi/WiN2d2+Z∝22∑NiPi1−Pi

Ultimately, we determined a sample size of 10,050 individuals, with 10,158 participants completing the survey. The distribution of participants across the cities is as follows: (1) Chuzhou: 1,171 participants; (2) Fuyang: 1,844 participants; (3) Chaohu: 2,742 participants; (4) Tongling: 2,558 participants; and (5) Anqing: 1,843 participants. To ensure methodological rigor and address potential biases, we implemented several measures during the administration of the questionnaires. Prior to distribution, participants were briefed on the importance of their honest responses for the study’s validity and were assured of the confidentiality of their answers. Prior to data collection, participants provided written consent. Questionnaires were administered under the supervision of well-trained graduates to minimize social desirability bias and ensure that the participants felt comfortable providing honest answers. Under the guidance of well-trained graduates, teenagers completed a self-administered questionnaire during class time. A total of 10,574 adolescents were admitted, the response rate was 96.07%, with 10,158 completed questionnaires. The respondents included 4,716 males (46.4%) and 5,442 females (53.6%), with an average age of 18.5 ± 1.384 years.

In our study of 10,156 adolescents, 24.5% were only children, predominantly from rural areas (76.1%). Parental education was predominantly low, with 48.1% of mothers and 22.9% of fathers having elementary education or less. Family income was spread, with 36.2% earning under 10,000 yuan and 15.9% above 30,000 yuan. Academic performance was medium for 67.7% of participants.

In this cross-sectional study, “Ethics Committee Approval” was obtained from Anhui Medical University Biomedical Ethics Committee with the number 2012570. The study followed the guidelines and principles set out in the Declaration of Helsinki, a set of ethical recommendations and principles for conducting research involving humans. Consent was obtained from the relevant school authorities, parents and students. The confidentiality of all information was carefully maintained by the research team, ensuring that no personal data was disclosed in the study findings.

### 2.2 Instruments

#### 2.2.1 Socio-demographic characteristics

The survey included socio-demographic characteristics and internet use situation of the participants. Age was categorized as follows: “1” = “<18 years old” and “2” = “≥18 years old.” Gender was coded as “1” for “male” and “2” for “female.” T Participants were asked if they were the only child, with “1” indicating “yes” and “2” indicating “no.” Residence was categorized as “1” for “rural” and “2” for “urban.” Parental educational background was coded as follows: “1” for “elementary school and below,” “2” for “middle school,” “3” for “secondary/technical school,” and “4” for “college and above.” Relationship with parents: Participants rated their relationship with their parents as “1” for “bad,” “2” for “general,” and “3” for “good.” Parental control was assessed on a scale from “1” for “little,” “2” for “general,” to “3” for “much.” Per capita annual household income was categorized as “1” for “<$1,505 (<10,000 yuan),” “2” for “$1,505–$3,011 (10,000–20,000 yuan),” “3” for “$3,011–$4,516 (20,000–30,000 yuan),” “4” for “$4,516–$6,022 (30,000–40,000 yuan),” and “5” for “>$6,022 (>40,000 yuan).” Participants rated their family conditions compared to their peers as “1” for “poor,” “2” for “below average,” “3” for “average,” “4” for “above average,” and “5” for “good.” Academic performance was categorized as “1” for “poor,” “2” for “medium,” and “3” for “good.” Additionally, participants were asked if they experienced bullying in schools in the last 12 months, with “1” indicating “yes” and “2” indicating “no.” Electronic bullying situation was assessed similarly, with “1” for “yes” and “2” for “no.” Furthermore, the average amount of time participants spent online each day was also collected.

#### 2.2.2 Young’s Internet Addiction Test

We evaluate the severity of internet addiction using the Internet Addiction Test (IAT) comprising 20 items ranked on a five-point scale (1–5). The total addiction score for each IAT (from 20 to 100) is the sum of the scores for 20 items. Scores less than 50 points were categorized as normal internet use, while scores between 50 and 80 indicated mild to moderate internet addiction, and scores exceeding 80 points were considered indicative of severe internet addiction (42). However, it is important to note that in this study, the primary focus was on using the scores to reflect the severity of internet addiction symptoms rather than strictly diagnosing individuals into specific categories. In this study, the internal validity coefficient (Cronbach’s alpha) was calculated as *α* = 0.886.

#### 2.2.3 The Rosenberg Self-Esteem Scale

The Rosenberg Self-Esteem Scale was used to measure adolescent self-esteem, consisting of 10 four-point items assessing current self-esteem (43). The reliability and structure validity of the scale was high (44). In this study, the internal validity coefficient (Cronbach’s alpha) was calculated as α = 0.799. The scale yields a single total score of self-esteem, ranging from 10 to 40. A cutoff score of less than 20 was defined as low self-esteem, scores between 20 and 30 were defined as normal, scores of 30 or higher were classified as high self-esteem (45).

#### 2.2.4 The Liebowitz Social Anxiety Scale

The Liebowitz Social Anxiety Scale (LSAS) was employed to assess the severity of anxiety symptoms related to the fear of being observed by others (46). The scale comprises two subscales: social anxiety and social avoidance, each consisting of 24 items, sorted on a 4-point scale ranging from 0 to 3. The total score for social anxiety is obtained by summing all items, ranging from 0 to 144. In this study, the internal validity α = 0.953.

### 2.3 Statistical analysis

SPSS 23.0 and AMOS 27.0 were used for all statistical analyses. Descriptive statistics were calculated for all variables, including median and quartiles for continuous variables such as IA, SES, SA item scores, and average daily online time. Classification variables were represented using numbers and percentages. Nonparametric tests were utilized to compare continuous variables between the internet addiction group (comprising mild and severe internet addiction) and the normal internet use group. Differences among the three groups for categorical variables were examined using the chi-square test. Employing Spearman correlation analysis to explore the relationship between internet addiction, self-esteem, and social anxiety. Statistical significance was set at a bilateral *p*-value of less than 0.05.

To control for potential confounding factors, we adjusted for demographic variables including age, gender, and parental education in our analysis. Using SEM to analyze the mediating role of social anxiety in the relationship between self-esteem and internet addiction. Model fit was assessed using the comparative fit index (CFI), the normal fit index (NFI), the goodness of fit index (GFI), and the root mean square error of approximation (RMSEA). Generally, CFI, NFI, and NNFI values exceeding 0.90, along with RMSEA values less than 0.08, are considered indicative of an acceptable model fit. Furthermore, we performed subgroup analyses by age and gender to explore the consistency of relationships across different demographics. Additionally, to address potential common method variance (CMV) concerns, we conducted Harman’s single-factor test.

## 3 Results

### 3.1 Descriptive statistics

Descriptive characteristics, internet addiction objects and average internet access of the study sample are shown in Table 1. Among the 10,158 participants, 4,716 were males (46.4%) and 5,442 were females (53.6%). The average age of the subjects was 19.00 years, ranging from 14 to 24 years old. The sample comprised 9,099 (89.6%) adolescents with normal internet usage, 1,038 (10.2%) adolescents with mild internet addiction, and 21 (0.2%) adolescents with severe internet addiction. Sociodemographic characteristics were compared among the normal internet usage group, moderate internet addiction group, and severe internet addiction group using nonparametric chi-square tests. The results are presented in Table 1.

**Table 1:** Descriptive characteristics of the study sample and internet addiction levels.

		Sample	Average internet use	Mild internet addiction	Severe internet addiction	*Z/χ* ^2^	*p*
All		10,158	9,099	1,038	21		
Age, %	14–18 years	1834 (18.1)	1,581 (86.2)	249 (13.6)	4 (0.2)	27.553	<0.001
	18–24 years	8,324 (81.9)	7,518 (90.3)	789 (9.5)	17 (0.2)		
Gender, %	Male	4,716 (46.4)	4,190 (88.8)	512 (10.9)	14 (0.3)	7.488	0.024
	Female	5,442 (53.6)	4,909 (90.2)	526 (9.7)	7 (0.1)		
Only child, %	Yes	2,487 (24.5)	2,201 (88.5)	280 (11.3)	6 (0.2)	4.100	0.129
	No	7,671 (75.5)	6,898 (89.9)	758 (9.9)	15 (0.2)		
Residence, %	Rural	7,729 (76.1)	6,948 (89.9)	767 (9.9)	14 (0.2)	4.146	0.126
	Urban	2,429 (23.9)	2,151 (88.6)	271 (11.2)	7 (0.3)		
Mother’s education background, %	Elementary school and blow	4,832 (48.1)	4,350 (48.4)	475 (46.3)	7 (35.0)	10.817	0.029
	Middle or secondary/technical school	4,946 (49.3)	4,424 (49.2)	510 (49.7)	12 (60.0)		
	College and above	263 (2.6)	221 (2.5)	41 (4.0)	1 (5.0)		
Father’s education background, %	Elementary school and blow	2,312 (22.9)	2076 (23.0)	231 (22.5)	5 (23.8)	4.382	0.357
	Middle or secondary/technical school	7,168 (71.1)	6,432 (71.2)	722 (70.2)	14 (66.7)		
	College and above	598 (5.9)	521 (5.8)	75 (7.3)	2 (9.5)		
Mother-adolescent relationship, %	Bad	756 (7.5)	623 (6.9)	130 (12.6)	3 (15.0)	45.334	<0.001
	Good	9,309 (92.5)	8,394 (93.1)	898 (87.4)	17 (85.0)		
Father-adolescent relationship, %	Bad	1,118 (11.1)	930 (10.3)	182 (17.8)	6 (28.6)	58.518	<0.001
	Good	8,963 (88.9)	8,105 (89.7)	843 (82.2)	15 (71.4)		
Parental control, %	Little	835 (8.2)	720 (7.9)	111 (10.6)	4 (19.2)	32.952	<0.001
	General	5,173 (50.9)	4,715 (51.8)	450 (43.4)	8 (38.1)		
	Much	4,150 (40.9)	3,664 (40.3)	477 (46.0)	9 (42.9)		
Annual family income (yuan), %	<10,000	3,677 (36.2)	3,340 (36.7)	330 (31.8)	7 (33.3)	16.357	0.003
	10,000 ~ 30,000	4,867 (47.9)	4,352 (47.8)	505 (48.7)	10 (47.6)		
	>30,000	1,614 (15.9)	1,407 (15.5)	203 (19.6)	4 (19.0)		
Academic performance, %	Poor	1899 (18.7)	1,572 (17.3)	320 (30.8)	7 (33.3)	118.745	<0.001
	Medium	6,876 (67.7)	6,264 (68.8)	603 (58.1)	9 (42.9)		
	Good	1,383 (13.6)	1,263 (13.9)	115 (11.1)	5 (23.8)		
Average daily online time, median (quartiles)	During summer holidays	4.00 (2.50, 6.00)	3.75 (2.50, 6.00)	2.00 (1.00, 4.00)	3.50 (2.00, 5.50)	245.205	<0.001
	During weekends	2.17 (1.17, 4.00)	5.00 (3.50, 8.00)	3.00 (2.00, 5.00)	5.00 (3.00, 8.00)	220.642	<0.001
	During weeks	4.00 (2.00, 6.00)	6.00 (5.25, 10.00)	4.00 (1.92, 5.50)	5.00 (4.75, 10.00)	194.666	<0.001

### 3.2 Factor structure and model fit analysis of scales

Exploratory Factor Analysis (EFA) conducted among our participants revealed a four-factor structure for the test of adolescent internet addiction, comprising IA1 (5 items: items 6, 1, 2, 8, 17), IA2 (5 items: items 15, 20, 11, 13, 16), IA3 (5 items: items 4, 9, 10, 7, 12), and IA4 (5 items). Subsequent Confirmatory Factor Analysis (CFA) demonstrated that the standard measurement model fit the data well, with the following fit indices: GFI = 0.991, CFI = 0.992, NFI = 0.992, and RMSEA = 0.091. Moreover, this study utilized structural equation modeling (SEM) to extract three factors from the scale: SES1 (5 items), SES2 (2 items), and SES3 (3 items). CFA demonstrated that the standard measurement model fit the data well: CFI = 0.997, GFI = 0.999, NFI = 0.997, RMSEA = 0.311. Regarding the Factor Structure Analysis of the Liebowitz Social Anxiety Scale (LSAS)，SEM was employed in the present study to extract four factors: SA1 (10 items), SA2 (6 items), SA3 (6 items), SA4 (2 items). CFA showed that the standard measurement model fit the data excellently, with the following fit indices: CFI = 1.000, GFI = 1.000, NFI = 1.000, RMSEA = 0.018.” Harman’s single-factor test indicated that the first factor in the IAT, SE, and SA accounted for 17.87, 26.26, and 22.50% of the total variance, respectively, all below the 40% threshold for CMV concern.

### 3.3 Differences in self-esteem and social anxiety between three groups

Table 2 displays the differences in self-esteem and social anxiety among mild internet addiction group, severe internet addiction group, and normal internet usage group. The self-esteem score (SES1: *Z =* 178.298, *p* < 0.001; SES2: *Z =* 45.163, *p* < 0.001; SES3: *Z =* 383.318, *p* < 0.001; the total score: *Z =* 385.136, *p* < 0.001) in both the mild internet addiction group and the severe internet addiction group were significantly lower compared to the normal internet usage group.

**Table 2:** Differences in self-esteem and social anxiety among internet use groups.

Scale	Normal internet use	Mild internet addiction	Severe internet addiction	*Z*	*p*	*η* ^2^
SES score, median (quartiles)						
SES1	8.00 (7.00, 9.00)	8.00 (8.00, 10.00)	9.00 (8.00, 11.00)	178.298	<0.001	0.165
SES2	6.00 (5.00, 6.00)	6.00 (6.00, 6.00)	6.00 (5.00, 7.00)	45.163	<0.001	0.075
SES3	6.00 (5.00, 7.00)	7.00 (6.00, 9.00)	9.00 (7.00, 11.00)	383.318	<0.001	0.211
Total SES scores	28.00 (26.00, 30.00)	26.00 (24.00, 28.00)	25.00 (20.50, 27.00)	385.136	<0.001	0.227
SA score, median (quartiles)						
SA1	32.00 (26.00, 39.00)	38.00 (31.00, 46.00)	43.00 (27.00, 65.00)	260.887	<0.001	0.239
SA2	21.00 (17.00, 26.00)	25.00 (20.00, 30.00)	28.00 (16.00, 40.50)	265.515	<0.001	0.213
SA3	20.00 (16.00, 24.00)	24.00 (19.00, 29.00)	29.00 (16.50, 37.50)	298.585	<0.001	0.214
SA4	6.00 (4.00, 8.00)	7.00 (5.00, 9.00)	8.00 (4.50, 13.00)	68.684	<0.001	0.094
Total SA scores	32.00 (18.00, 48.00)	47.00 (30.00, 64.00)	61.00 (18.50, 104.00)	302.856	<0.001	0.256

SES1, SES2, SES3 represent the three factors extracted from the scale using structural equation modeling. SA1, SA2, SA3, SA4 represent the four factors extracted from the scale using structural equation modeling.

In the survey of social anxiety, the scores of both the mild internet addiction group and severe internet addiction group were higher than those of the normal internet usage group (SA1: *Z =* 260.887, *p* < 0.001; SA2: *Z =* 265.515, *p* < 0.001; SA3: *Z =* 298.585, *p* < 0.001; SA4: *Z =* 68.684, *p* < 0.001; the total score: *Z =* 302.856, *p* < 0.001). According to the scale of social anxiety, 672 (64.7%) individuals in the mild internet addiction group and 13 (61.9%) in the severe internet addiction group experienced social anxiety. The prevalence of social anxiety in the mild internet addiction group and severe internet addiction group were higher than that in the normal internet use group (*χ*^2^ *=* 234.363, *p* < 0.001).

### 3.4 The association between internet addiction, self-esteem and social anxiety

As shown in Table 3, the IA score demonstrated a negative association with self-esteem (IA with SES1: *r* = 0.222, *p* < 0.01; IA with SES2: *r* = 0.112, *p* < 0.01; IA with SES3: *r* = 0.267, *p* < 0.01), while a positive association was observed with social anxiety (IAT with SA1: *r* = 0.317, *p* < 0.01; IA with SA2: *r* = 0.347, *p* < 0.01; IA with SA3: *r* = 0.337, *p* < 0.01; IA with SA4: *r* = 0.153, *p* < 0.01).

**Table 3:** The correlations of internet addiction, self-esteem, and social anxiety.

	IA score	SES1	SES2	SES3	SA1	SA2	SA3	SA4
IA score	1							
SES1	0.222^**^	1						
SES2	0.112^**^	0.011	1					
SES3	0.267^**^	0.395^**^	0.297^**^	1				
SA1	0.317^**^	0.326^**^	0.132^**^	0.251^**^	1			
SA2	0.347^**^	0.341^**^	0.130^**^	0.252^**^	0.788^**^	1		
SA3	0.337^**^	0.326^**^	0.124^**^	0.254^**^	0.810^**^	0.793^**^	1	
SA4	0.153^**^	0.150^**^	0.064^**^	0.105^**^	0.510^**^	0.485^**^	0.496^**^	1

SES1, SES2, SES3 represent the three factors extracted from the scale using structural equation modeling. SA1, SA2, SA3, SA4 represent the four factors extracted from the scale using structural equation modeling. **: *p* < 0.01.

### 3.5 Measurement and structural model for detecting mediating effects

**Figure 1:**
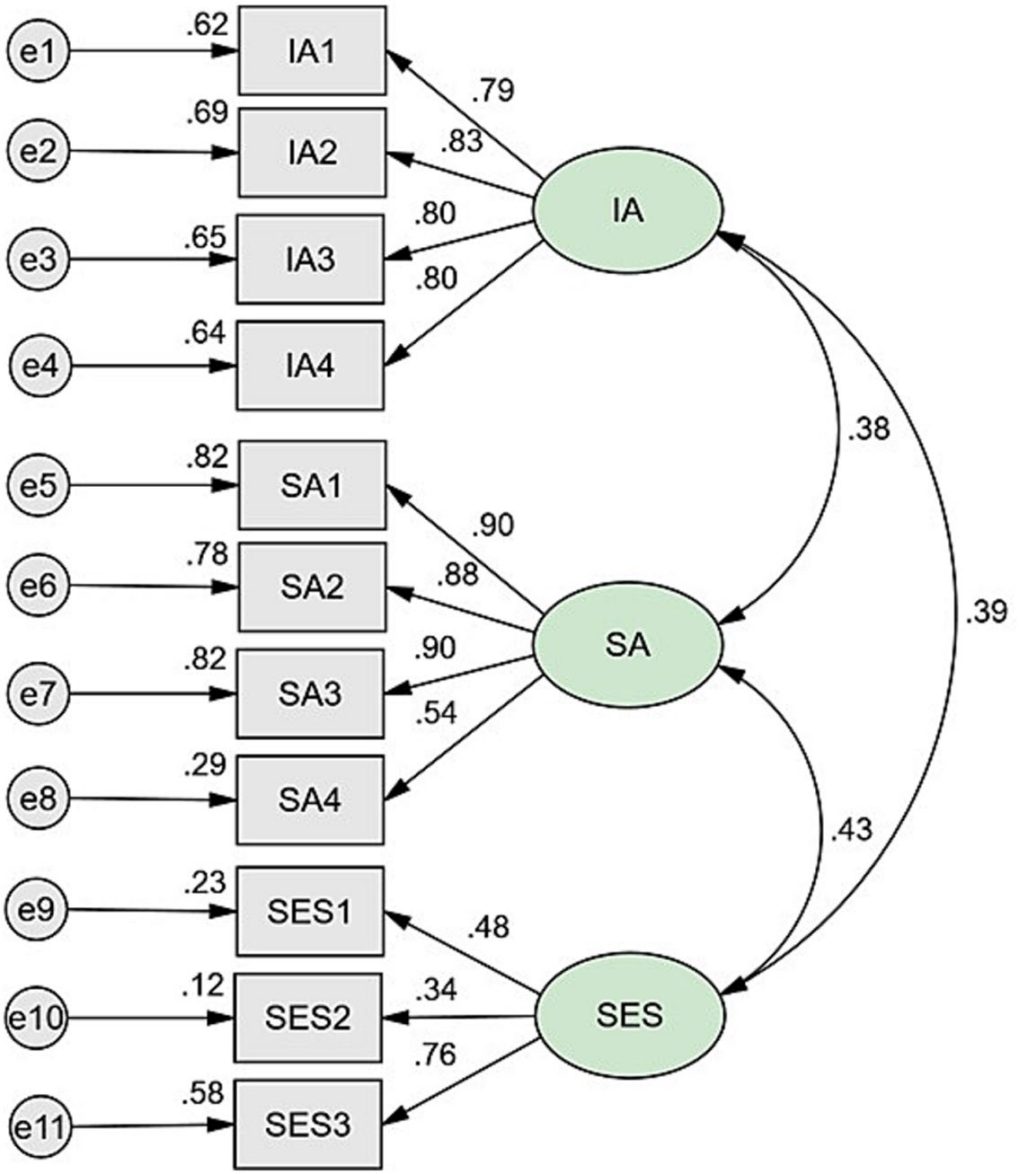
The confirmatory factor analysis of the measurement model for the accept fit of the data.

The preliminary test of the measurement model indicates a good fit with the actual data. In this study, the measurement model was initially evaluated for its fit to the data through a CFA. As illustrated in Figure 1, CFI = 0.969, NFI = 0.968, GFI = 0.971, RMSEA = 0.064. The loading of the 11 measurement variables on the 3 latent variables was statistically significant (*p* < 0.001). The factor loading of the measured variables on the latent variables revealed significant associations, with self-esteem being significantly related to internet addiction (*r* = 0.523, *p* < 0.01) and social anxiety (*r* = 0.410, *p* < 0.01). Moreover, social anxiety could predict internet addiction (*r* = 0.506, *p* < 0.01). The three latent variables of internet addiction, social anxiety, and self-esteem were measured using observed variables.

**Figure 2:**
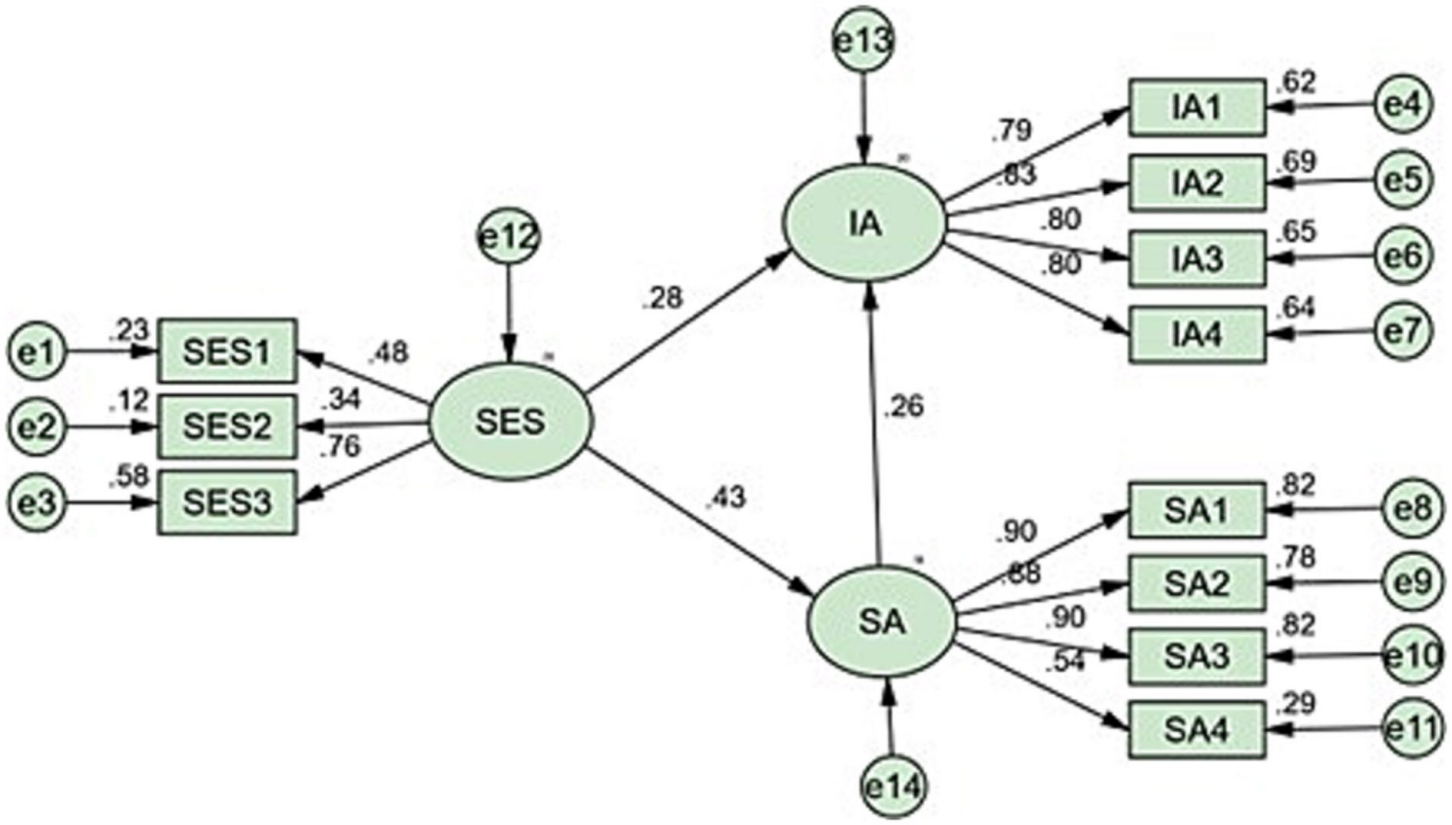
Structural equation model of the relationships between self-esteem, social anxiety and internet addiction. Circles represent latent variables and rectangles represent measured variables.

As shown in Figure 2, a mediating model was established between self-esteem and internet addiction, with social anxiety serving as the mediating variable. Internet addiction was considered the dependent variable, while self-esteem was treated as the independent variable. The results of the structural model indicated a good fit with the data across all criteria (CFA = 0.969, NFI = 0.968, GFI = 0.971, RMSEA = 0.064). The standardized path coefficient from self-esteem to internet addiction was 0.28 (*t* = 16.899, *p* < 0.01), and the standardized path coefficient from social anxiety to internet addiction was 0.26 (*t* = 19.592, *p* < 0.01). The indirect effect of self-esteem on social addiction through social anxiety was 0.11 (*p* < 0.01), accounting for 28.35% of the total effect, and the total impact of self-esteem on internet addiction was 0.278 (*p* < 0.01). Subgroup analyses were conducted to assess the robustness of the relationship between self-esteem and internet addiction through social anxiety across different demographics. These findings suggest that the relationship between self-esteem and internet addiction, mediated by social anxiety, is consistent across different age groups and genders, see Table 4.

**Table 4:** The model fit and the effects of self-esteem on internet addiction through social anxiety in different subgroups.

		*χ* ^2^	CFI	NFI	GFI	RMSEA	Direct effects	Indirect effects	Total effects
Age	14–18 years	317.327	0.970	0.966	0.970	0.061	0.473^**^	0.164^**^	0.637^**^
	18–24 years	729.240	0.975	0.973	0.977	0.056	0.517^**^	0.227^**^	0.744**
Gender	Male	1000.231	0.964	0.963	0.965	0.070	0.277^**^	0.108^**^	0.385^**^
	Female	729.240	0.975	0.973	0.977	0.056	0.517^**^	0.227^**^	0.744^**^

**: *p* < 0.01.

## 4 Discussion

In our study, we investigated the prevalence of internet addiction among Chinese teenagers and its correlation with self-esteem and social anxiety. Utilizing the Young Internet Addiction Test, we found that 10.40% of participants exhibited signs of internet addiction, aligning with recent cross-sectional studies in China that highlight its growing concern (47, 48). Our findings underscore the complex interplay between self-esteem and internet addiction, with social anxiety identified as a pivotal mediator. This suggests that adolescents with lower self-esteem may be more susceptible to developing internet addiction, potentially using the internet as a maladaptive coping strategy that could be further complicated by social anxiety.

### 4.1 The status quo and prevalence of internet addiction

The study identified significant differences among the mild internet addiction group, severe internet addiction group, and normal internet use group concerning age, gender, maternal education, parent–child relationship, parental control, family annual income, academic performance, and average online time. In terms of age, a higher prevalence of internet addiction was observed among younger (<18 years) adolescents compared to their older counterparts (≥18 years), with significant differences observed between the normal internet usage group, mild internet usage group, and severe internet usage group. It is posited that younger teenagers may exhibit poorer self-control, potentially contributing to their heightened susceptibility to internet addiction. This finding diverges from the study by Ko et al. (49), which involved 2,293 seventh-grade adolescents across 10 junior high schools in Taiwan, and concluded that there was no difference in age between the internet addiction group and the non-internet addiction group (49). This result is in line with Shao et al.'s 2018 meta-analysis on the prevalence of internet addiction among Chinese college students, which found the detection rate was higher in male students (16%) than female students (8%) (50). In contrast, Kang et al.'s 2023 cross-sectional survey of 5,824 college students from 8 universities in China also pointed out that the prevalence of IA was slightly higher in females than in males (51). This discrepancy may be related to the increased demand for online social interaction, such as through WeChat, and online shopping, such as on Taobao, among females (52).

Several studies, including our, have suggested that annual family income serves as a potential risk factor of internet addiction (53, 54). This association may be attributed to the increased opportunities for teenagers from financially stable families to engage with the internet. The results of this study also reveal a significant relationship between internet addiction and parental control, which aligns with the findings of Wu et al. 2014 cross-sectional study conducted in vocational high schools and higher vocational colleges in Anhui Province, indicating that parental control is an important risk factor for adolescent internet addiction (55). In the process of youth transformation and development, parental monitoring or guidance of leisure activities is particularly essential. Additionally, disparities in mothers’ education levels were observed among the normal internet use group, mild internet addiction group, and severe internet addiction group, aligning with studies such as Wang W et al. which investigated 998 middle school students across five schools in Wuhan and Shanghai, China, and found consistent results. However, Seyrek et al. (56) found no significant differences in the educational levels of mothers among Turkish adolescents categorized into normal internet users, those with mild internet addiction, and those with severe internet addiction, based on a cross-sectional study involving 468 students from private, vocational, and public schools (56). Furthermore, our study highlighted the significance of the parent-adolescent relationship as a risk factor for adolescent internet addiction, consistent with findings from previous research (57, 58). In a sense, it is implying that elevated parent-juvenile conflict was associated with a higher prevalence of internet addiction among adolescents, emphasizing the importance of fostering a harmonious family environment for teenage well-being. Moreover, adolescents’ academic performance emerged as a risk factor for internet addiction, with a relatively low incidence observed among individuals with commendable academic achievements, corroborating findings from various studies (59, 60). Lastly, our study indicated a significant relationship between increased average daily online time and the development of internet addiction, consistent with previous research findings (42, 61).

We acknowledge that the sample size for severe internet addiction is relatively small (21 individuals), which may limit our ability to conduct an in-depth analysis of this specific group. Nonetheless, it is important to note that the prevalence of severe internet addiction in the general population is inherently low, as supported by existing literature (62, 63). Future research should delve into the longitudinal progression of internet addiction among adolescents, particularly in vocational school settings. It should also examine how gender-specific factors influence the development of internet addiction. Additionally, the role of family dynamics, including parental control and family income, should be further explored to inform potential interventions. Educational strategies to bolster academic performance and mitigate the risk of internet addiction also warrant investigation. Lastly, the influence of socio-economic status on internet addiction should be scrutinized to guide policy and intervention efforts.

### 4.2 Differences in stressful self-esteem and social anxiety

Adolescents in vocational schools are likely to suffer from inferiority due to their poor academic performance. Low levels of self-esteem, characterized by negative self-evaluation and social anxiety, are prevalent among this demographic. In this study, self-esteem, self-doubt, and self-denial were used to evaluate adolescents’ self-esteem. Inaccurate self-recognition among adolescents can precipitate various negative outcomes. Our findings revealed that levels of self-esteem were higher in the normal internet usage group compared to the mild and severe internet addiction groups. This aligns with the results of Shi et al.’s relatively large-scale survey of 3,065 students across six high schools in Shanghai, China, which demonstrated a significant negative correlation between internet addiction and self-esteem (64). Similarly, Dai et al.’s survey of 1,912 students in 50 classes from the East, South, and Central regions of mainland China using a hierarchical linear model also indicated that students with low self-esteem are at a higher risk of IA compared to those with high self-esteem (65). Perspective studies proposed that low self-esteem may contribute to internet addiction by offering individuals a virtual refuge to fulfill their internal needs (66). From a CBT perspective, low self-esteem can lead to maladaptive coping mechanisms, such as excessive internet use, as a way to escape negative self-perceptions and social anxiety. This aligns with perspective studies that suggest low self-esteem may contribute to internet addiction by offering a virtual refuge to fulfill internal needs (67).

Tan et al., utilizing a generalised mixed linear model, discovered in their study of 4,647 university students across 42 institutions in 20 major Chinese cities that social anxiety has a direct impact on internet addiction among college students, and it emerges as a common mental health issue for adolescents dealing with internet addiction (68). Our study corroborates this trend, indicating a heightened likelihood of social anxiety among internet-addicted teenagers. Specifically, both the mild and severe internet addiction groups exhibited higher prevalence rates of social anxiety compared to the normal internet usage group. Moreover, the Internet Addiction Test (IAT) score positively correlated with levels of social anxiety. CBT would suggest that social anxiety leads to avoidance behavior and that the internet provides a platform for adolescents who may overuse the internet to cope with social anxiety. This avoidance behavior reinforces negative thinking patterns and leads to a vicious cycle of increased internet use and social anxiety (69).

The findings of this study highlight the critical role of low self-esteem and social anxiety in the development of internet addiction among adolescents. To address these issues, CBT offers a robust framework for intervention. For adolescents with low self-esteem, self-compassion training could be an effective intervention. This approach encourages individuals to adopt a kinder and more accepting attitude towards themselves, which may counteract negative self-evaluations (70). Given that our study found lower self-esteem levels in the internet addiction groups, self-compassion training could help these adolescents develop a healthier self-concept and reduce their reliance on the internet as a source of self-worth. By fostering self-compassion, adolescents may learn to treat themselves with kindness and recognize that their self-worth does not depend on external validation (71). This shift in perspective could potentially mitigate the need for excessive internet use as a means of seeking validation and, in turn, reduce the risk of internet addiction.” In addition, exploring the therapeutic potential of addressing social anxiety could provide insights into reducing rates of internet addiction. Exposure techniques within CBT frameworks could be particularly effective in treating social anxiety (72). These techniques involve using exposure therapy to gradually expose adolescents to feared social situations in a controlled manner. Lin et al. found that arousal feedback–based exposure therapy is effective in alleviating symptoms of social anxiety, particularly public speaking anxiety (73). This suggests that such exposure techniques could provide a promising approach to addressing social anxiety, reducing reliance on the internet as a coping mechanism.

### 4.3 The mediation model of internet addiction

To date, few studies have explored the role of self-esteem in the development of internet addiction or the construction of models elucidating the pathways from self-esteem to internet addiction. This study assumes that social anxiety serves as a mediator in the relationship between self-esteem and internet addiction, employing structural equation modeling to test hypotheses. The fitted statistics of both the measurement model and the structural model exhibit good fit with the data (see Figure 1). The relationship between internet addiction and self-esteem (*r* = 0.39) is slightly stronger than the association between social anxiety and internet addiction (*r* = 0.38). From the results of the structural model, the standardized path coefficients including self-esteem, social anxiety (*β* = 0.43) and social anxiety internet addiction (*β* = 0.26) were statistically significant. These findings from the structural model provide support for the hypothesis that self-esteem indirectly influences internet addiction through social anxiety. The results show that there is a significant positive correlation between social anxiety and internet addiction. The path from internet addiction to self-esteem (*β* = 0.28) is similarly significant, indicating the presence of the relationship between self-esteem and internet addiction. The indirect effect of self-esteem on internet addiction through social anxiety was found to be 0.11, while the total impact of life events on internet addiction was 0.39. The percentage of direct influence and indirect influence was calculated as 71.65 and 28.35%, respectively. The value of the standardized path coefficient indicates that the self-esteem directly contributes to internet addiction, and also indirectly leads to internet addiction through social anxiety. In addition, it was observed that the direct effect was greater than the indirect effect. Ultimately, the findings support the hypothesis that self-esteem can indirectly lead to internet addiction through social anxiety among adolescents.

It is worth noting that the direct effect of self-esteem on internet addiction was stronger than the indirect effect through social anxiety. This finding may be attributed to the multifaceted nature of self-esteem and its broader impact on adolescent behavior. Self-esteem is a fundamental psychological construct that influences various aspects of an individual’s life, including their motivation, goal-setting, and coping strategies. Adolescents with lower self-esteem may be more prone to internet addiction (74) not only because of social anxiety but also due to other factors such as a lack of self-efficacy, negative self-perception (17), and the search for validation through online activities (75). In this context, the direct effect of self-esteem on internet addiction may reflect a more general tendency for individuals with low self-esteem to engage in maladaptive behaviors as a means of self-regulation. Moreover, the partial mediation effect indicates that while social anxiety is an important mediator, it is not the sole pathway through which self-esteem influences internet addiction. Other potential mediators, such as negative affect and the negative semantic dimensions of relationship satisfaction (76), may also play a role in this relationship. Future research could explore these additional pathways to provide a more comprehensive understanding of the mechanisms underlying the association between self-esteem and internet addiction.

Our research integrates cognitive-behavioral theories to propose that self-esteem is a fundamental determinant that may predispose individuals to internet addiction. Social anxiety is hypothesized to mediate this relationship, potentially exacerbating the risk of developing internet addiction. These results contribute to a deeper understanding of the psychological mechanisms underlying internet addiction and suggest that interventions targeting self-esteem and social anxiety could be pivotal in mitigating the risk of internet addiction among adolescents. The bidirectional relationship between self-esteem and internet addiction, as well as the mediating role of social anxiety, underscores the complexity of this issue and the need for a multifaceted approach to prevention and treatment strategies.

### 4.4 Limitations

The present study acknowledges several inherent limitations that warrant further elaboration. Primarily, the research employs a cross-sectional design, which inherently restricts the capacity to infer causality between the variables under observation. This design limits our understanding of the temporal sequence of events as it captures data at a single point in time. Additionally, measures that rely on self-report introduce potential biases such as social desirability bias and recall bias. Though the results of Harman’s single-factor test, with percentages below 40% for all factors, indicate that CMV is not a significant concern in our study, participants may wish to present a favorable image of themselves or have difficulty accurately recalling past events, which may affect the accuracy and reliability of the data collected. Moreover, while the sample size encompasses Chinese vocational school students from five different cities/regions within Anhui province, it may not be fully representative of all Chinese vocational school students. Therefore, a larger and more diverse sample is required to validate the current hypothesis.

## 5 Conclusion

This study provides a comprehensive examination of the intricate relationship between self-esteem, social anxiety, and internet addiction among adolescents. Our findings indicate that self-esteem is both directly and indirectly linked to internet addiction, with social anxiety serving as a significant mediator. This suggests that adolescents with lower self-esteem may be more susceptible to internet addiction, potentially due to the role of social anxiety as an intermediary factor. The implications of these findings extend beyond the individual level, offering valuable insights into the societal factors that contribute to the prevalence of internet addiction among young people. By understanding the role of self-esteem and social anxiety, we can better appreciate the complex interplay of psychological and behavioral factors in the development of internet addiction. This has broader societal implications, suggesting that interventions aimed at enhancing self-esteem and reducing social anxiety could be instrumental in curbing the incidence of internet addiction among adolescents.

For practitioners, it is crucial to create an educational and social environment that nurtures self-esteem and provides effective coping mechanisms for social anxiety. Schools and community programs could integrate modules that focus on developing resilience, emotional intelligence, and healthy online behaviors. Furthermore, mental health professionals should consider these factors when designing intervention strategies, potentially leading to more targeted and effective approaches to prevent and treat internet addiction in adolescents.

## Data Availability

The raw data supporting the conclusions of this article will be made available by the authors, without undue reservation.
